# RE: “Estimated Rate of Reactivation of Latent Tuberculosis Infection in the United States, Overall and by Population Subgroup”

**DOI:** 10.1093/aje/kwu187

**Published:** 2014-07-18

**Authors:** R. M. G. J. Houben, T. A. Yates, D. A. J. Moore, T. D. McHugh, M. Lipman, E. Vynnycky

**Affiliations:** 1 North London TB Journal Club, London, United Kingdom; 2 TB Modelling Group, London School of Hygiene and Tropical Medicine, London, United Kingdom; 3 TB Centre, London School of Hygiene and Tropical Medicine, London, United Kingdom; 4 Research Department of Infection and Population Health, University College London, London, United Kingdom; 5 Centre for Clinical Microbiology, Division of Infection and Immunity, University College London, London, United Kingdom; 6 Department of Medicine, University College London, London, United Kingdom; 7 Statistics, Modelling and Economics Department, Public Health England, London, United Kingdom

Tuberculosis (TB) disease can occur soon after new infection (or reinfection) or many years thereafter through reactivation of latent infection (1). Reliable estimates of rates of reactivation are needed to predict the impact of interventions, particularly in low-burden, high-income settings where there may be little ongoing transmission.

We welcome Shea et al.'s (2) recent attempt to directly calculate this rate for the whole of the United States using empirical data. This could have permitted validation of currently used estimates, obtained from mathematical models that fit to country-level historical data (3). Shea et al. reported differential TB reactivation rates by place of birth and human immunodeficiency virus (HIV) status. If valid, these estimates would also be valuable. However, we have concerns about the assumptions used to derive these metrics from the limited data available. To calculate the rate of TB reactivation in the United States, Shea et al. divided estimates of the number of cases that they attributed to reactivation by estimates of the number of persons considered at risk of reactivation (2).

For the numerator (cases of reactivation TB), they used TB isolates from 2006–2008 with a unique genotype within the national genotyping database. Here, there were substantial missing data, that is, TB cases without a genotype. Only 57% of all TB cases (73% of culture-positive cases) reported to the Centers for Disease Control and Prevention were genotyped. Results from modeling studies (4, 5) have shown that the chance that cases will be incorrectly attributed to reactivation is increased if only a proportion of all cases are genotyped. This is a particular problem when cluster sizes are small—which is likely here, given the low-burden setting. The relatively short sampling frame would also result in overestimation of the number of cases due to reactivation (4, 5), as might the authors' assumption that transmission cannot occur between persons living more than 50 km apart. Based on findings by Glynn et al. (5), the missing data might result in the proportion of cases attributable to reactivation being overestimated by as much as 60%. The use of a relatively nondiscriminatory typing method (12-locus MIRU-VNTR [mycobacterial interspersed repetitive units–variable number of tandem repeats] (6)) may have resulted in some bias in the other direction but adds further uncertainty to these estimates. The authors applied the proportion reactivated, calculated from the observed data, to persons with missing genotypes, thus amplifying any misclassification.

For the denominator, Shea et al. estimated the prevalence of latent infection from 7,386 persons who received a tuberculin skin test (TST) in cross-sectional health surveys in 1999 and 2000, and then extrapolated to estimate the prevalence of latent infection for the entire US population (over 300 million people) (2). While a 10-mm TST cutoff for all populations is convenient, it feels simplistic given the known variation in nontuberculous mycobacteria exposure both between countries and within the United States (7) and the likely high coverage of Bacillus Calmette-Guérin vaccination in the foreign-born population.

The authors also present reactivation rates by HIV status, under the assumption that the prevalence of latent infection does not differ by HIV status (2). This is inappropriate, given the fact that in high-income countries, these infections are often co-located in disadvantaged communities (8).

When presenting estimates of reactivation rates among HIV-negative persons, the authors might have attempted to quantify the impact of their assumptions with sensitivity analyses. These could have included allowing the TST cutpoint to vary (e.g., by place of birth) and adjusting the estimated number of reactivation cases to account for potential sampling bias. With regard to HIV, we agree with the authors that better understanding of the interaction between HIV infection and TB reactivation is needed, but the available data do not allow valid calculation of these rates.

In summary, the questions addressed by Shea et al. (2) are important, but the conclusions drawn should be more cautious.
